# Differentiating Canine Chronic Inflammatory Enteropathies Using Faecal Amino Acid Profiles: Potential and Limitations

**DOI:** 10.3390/ani15081185

**Published:** 2025-04-21

**Authors:** Cristina Higueras, Claudia Ruiz-Capillas, Ana Herrero, Angel Sainz, Mercedes García-Sancho, Fernando Rodríguez-Franco, Mar Larrosa, Ana I. Rey

**Affiliations:** 1 Animal Nutrition, Department of Animal Production, Faculty of Veterinary Medicine, Complutense University of Madrid, Avda. Puerta de Hierro s/n., 28040 Madrid, Spain; crhiguer@ucm.es; 2 Institute of Science and Technology of Food and Nutrition, Spanish National Research Council (CSIC), 28040 Madrid, Spain; 3 Department of Medicine and Animal Surgery, Faculty of Veterinary Medicine, Complutense University of Madrid, Avda. Puerta de Hierro s/n., 28040 Madrid, Spain; 4 Department of Nutrition and Food Science, Faculty of Pharmacy, Complutense University of Madrid, Plaza Ramón y Cajal s/n., 28040 Madrid, Spain

**Keywords:** aromatic amino acids, branched-chain amino acids, tyrosine, threonine, gut health, chronic inflammatory enteropathies, dogs

## Abstract

The aims of this study were to characterise the faecal amino acid profile of dogs with different chronic digestive diseases (food-responsive enteropathy (FRE), immunosuppressant-responsive enteropathy (IRE)) prior to dietary change, and *Giardia* infection (GIA), compared to healthy control (HC), and to evaluate their discriminating potential. The HC group presented lower faecal tyrosine (Tyr) and aromatic amino acids (AAAs) compared to FRE or IRE dogs (*p* = 0.0001). Additionally, the HC group had lower levels of threonine (Thr) (*p* = 0.0005) than the IRE group, while FRE dogs showed intermediate values. No statistically significant differences in faecal amino acids were observed between FRE and IRE dogs. In contrast, the GIA group had higher faecal amino acid values (except glutamic acid (Glu)) compared to the other dogs. The most determinant variables contributing to the discriminant functions were Tyr, Glu, arginine, and phenylalanine. Validation results of the discriminant functions showed that 44% of stool samples were misclassified, resulting in a 56% success rate. The faecal amino acid profile did not accurately distinguish FRE from IRE dogs; however, faecal excretion of AAs was generally higher in dogs with GIA.

## Simple Summary

This study explores the potential of faecal amino acids as non-invasive biomarkers for distinguishing different forms of canine chronic inflammatory enteropathies. The findings suggest that faecal amino acid profiles, particularly threonine and aromatic amino acids such as tyrosine, could serve as indicators of chronic digestive disorders. However, the faecal amino acid profile alone cannot fully differentiate dogs that respond to diet from those that do not. Dogs with clinical signs but infected with *Giardia* spp. show significantly greater excretion of faecal amino acids compared to the others.

## 1. Introduction

Chronic inflammatory enteropathies (CIEs) in dogs are characterised by a multifactorial origin, in which an exacerbated immune response, environmental factors (such as diet and microbiota), and a genetic predisposition are thought to play a role [1,2]. In this context, it has been observed that certain food components can affect the integrity of the intestinal epithelium, which is essential for maintaining homeostasis, gut microbiota balance, and the mucosal immune system [3]. Regarding protein, it remains unclear whether it exacerbates inflammation or, conversely, has positive effects through bioactive peptides or amino acids. These components may influence intestinal homeostasis, modulate immune responses, or impact intestinal tight junction proteins [4,5].

Thus, diets based on easily digestible protein or novel protein are commonly used in veterinary medicine for CIEs, although a wide variability in response to dietary treatment has been observed [1]. Dogs responding to diet (FRE) typically experience clinical remission after adopting these specialised diets, whereas non-responders require further invasive diagnostic tests that reveal an inflammatory process in the intestinal mucosa and treatment with immunosuppressants (IRE). The reason for these differing dietary responses remains unknown [1]. Recent studies suggest that dietary protein content may influence gut bacterial diversity, and microbiome changes may significantly affect the host’s amino acid metabolism [6].

Considering that these are idiopathic diseases whose diagnosis is based on the response to diet, in recent years, there has been an intensified search for compounds that allow for faster differentiation. Thus, in human medicine, significant changes in circulating amino acids (AAs) have been observed in individuals with inflammatory bowel disease (IBD) compared to healthy controls [7,8]. These changes have been considered useful for diagnosis, monitoring and understanding the pathogenesis of these diseases. In veterinary medicine, studies have also shown differences in the AA profiles of dogs with gastrointestinal diseases compared to healthy dogs. Thus, Tamura et al. [9] observed decreased serum AA levels and correlated serine (Ser) with the canine chronic enteropathy clinical activity index (CCECAI). Similarly, Benvenuti et al. [10] reported significant changes in serum phenylalanine (Phe), tryptophan (Trp), and histidine (His) levels based on the severity of the enteropathy. Other studies have evaluated serum AA concentrations in protein-losing enteropathy [11]. In cats with chronic gastrointestinal diseases, plasma AA levels have been considered more sensitive nutritional markers than other parameters, such as total proteins or albumin levels [12].

Most research on CIEs in animals has focused on blood AA levels, with little information on the potential use of non-invasive methods, such as the faecal amino acid profile to differentiate these diseases. In humans, Bosch et al. [13] highlighted the high potential of faecal AAs as novel non-invasive, low-cost biomarkers for diagnosing pediatric IBD. Recent studies on dogs with CIEs versus controls have also reported increased faecal Trp levels [14]. Moreover, Higueras et al. [15] observed some variations in the faecal AA profile, although the most notable changes occurred in serum AAs; this study, however, only compared FRE dogs to healthy controls.

To date, no study has evaluated the changes in faecal AA profile in dogs with different CIEs or the potential of these compounds as discriminating factors for correct diagnosis and tailored dietary strategies. Given that serum and faecal AA composition reflects the host’s metabolic state and possible alterations in gut microbiota [6], their study deserves further attention.

On the other hand, among the diseases that can potentially cause chronic diarrhea, and other associated clinical signs are parasitic infections. These processes must be diagnosed first in order to determine whether we are dealing with a parasitic problem or, on the contrary, whether it is an inflammatory process with idiopathic characteristics such as CIEs. *Giardia* (GIA) is one of the most frequent protozoan infections affecting both humans and animals [16]. However, there is a lack of information on the changes that this parasite can produce in the faecal AA profile or how they differ from those in CIEs.

Thus, the aim of this study was, firstly, to characterise the faecal amino acid profile of dogs with different chronic digestive diseases (FRE, IRE), prior to dietary change and GIA in comparison to healthy individuals; and secondly, to evaluate their discriminating potential and clinical utility.

## 2. Materials and Methods

The procedures and protocols outlined in this study received approval from the Animal Research Committee of the Complutense Veterinary Medicine Teaching Hospital (CVMTH) under reference number 11/2021 (approval date: 26 May 2021). All dog owners provided informed consent for their pets’ participation.

### 2.1. Experimental Design, Animal Signalment and Diets

This study involved 62 sick dogs presenting with gastrointestinal signs, referred to the CVMTH between January 2022 and March 2023. To be included, dogs had to exhibit digestive signs such as vomiting, diarrhoea, weight loss, and/or anorexia/hyporexia for at least three weeks. A comprehensive diagnostic process was carried out, which included, at least, physical exams, faecal parasite screening, blood analysis, serum biochemistry including trypsin-like immunoreactivity (TLI) and cortisol, and ultrasound. In addition, each dog underwent trials with at least two elimination diets featuring novel or hydrolysed proteins [17]. For those unresponsive to dietary changes, endoscopic biopsies and histological samples were taken for further evaluation.

The dogs were grouped into three diagnostic categories: thirty-five were identified with FRE due to their positive response to dietary changes within one month. Eighteen were diagnosed with IRE, characterised by a lack of response to diet alone, improvement with immunosuppressants, and histological signs of inflammation. Nine dogs were confirmed to have GIA, verified through modified Telemann and Merthiolate iodine formaldehyde tests (ESCCAP), as well as a favourable response to specific treatment against this parasite. Other potential causes of gastrointestinal inflammation or systemic diseases were ruled out to refine the diagnoses.

Dogs with FRE consisted of 16 females (7 intact, 9 spayed) and 19 males (8 intact, 11 neutered). The median age in this group was 3.25 ± 3.50 years (range: 1–12 years), and the median body weight was 10.60 ± 12.73 kg (range: 2.60–45.50 kg). The breeds included 5 crossbreds and 30 purebreds. In the IRE group, there were 12 females (1 intact, 11 spayed) and 6 males (5 intact, 1 neutered), with a median age of 7 ± 2.95 years (range: 1.70–11 years) and a median body weight of 7.32 ± 9.45 kg (range: 1.19–36.20 kg). There were 5 crossbred and 13 purebred dogs. The GIA group consisted of 2 females (1 intact, 1 spayed) and 7 males (4 intact, 3 neutered). The median age of this group was 8 ± 4.38 years (range: 1–12 years), and the median body weight was 12.60 ± 7.43 kg (range: 2.70–26.30 kg). There were 1 crossbred and 8 purebred dogs.

Alongside the affected dogs, a control group of 22 healthy control dogs (HC) was included as a baseline. These control dogs were selected based on normal physical exams, blood tests, and the absence of clinical signs for at least four months. Dogs with asymptomatic chronic diseases were excluded from the control group. The control group consisted of 13 females (4 intact, 9 spayed) and 9 males (6 intact, 3 neutered), with a median age of 5 ± 2.96 years (range: 1–12 years) and a median body weight of 19.40 ± 15.02 kg (range: 7.50–55 kg). Regarding breed, 15 dogs were purebred and 7 were crossbred.

Faecal samples were collected by owners over three consecutive days using sterile containers. Two of the samples were refrigerated until the clinical examination day, while the third one was collected on the morning of the visit. Once delivered by the owner, the fresh sample was immediately split into two containers and frozen at −20 °C for amino acid determinations. For testing, two refrigerated samples and the other fresh sample were used for parasite detection. Faecal consistency was assessed using the Purina^®^ Faecal Scoring Chart (Société des Produits Nestlé S.A., Vevey, Switzerland), which ranges from one (very hard and dry) to seven (watery). Additional data collected during the visit included age, sex, fertility status, breed, body weight, body condition score (BCS), muscle condition score (MCS), and the canine inflammatory bowel disease activity index to assess clinical symptom severity (CIBDAI) [18].

Prior to the dietary trial, information regarding the dogs’ commercial diets was gathered for every dog included in the study (Table 1). In all diets, the main source of protein was of animal origin. Those animals receiving a homemade diet were excluded from the study.

### 2.2. Measurement of Amino Acids in Faecal Samples

Lyophilised samples (0.1 g) (Lyoquest, Telstar, Tarrasa, Spain) were placed in screw-capped glass tubes and hydrolysed with 25 mL of 6 M HCl. These tubes were then flushed with N_2_ and heated to 110 °C for 22 h. Then, samples (after cooling at room temperature) were filtered through filter paper to a beaker, and the pH was adjusted to 5.6 by the addition of NaOH solution (pH meter Euthech, Thermo Scientific, Waltham, MA, USA). The solution was placed in a 100 mL volumetric flask and levelled up to that volume. Then, 20 mL were collected with a syringe and filtered using a Sep-pak silica cartridge. Subsequently, 2 mL of the sample extract was isolated in a vial and stored at −20 °C.

The amino acid content was determined using a Biochrom 20 amino acid analyser (Amersham Pharmacia Biotech., Biocom, Uppsala, Sweden), using the methodology of amino acid analysis based on ion exchange chromatography and post-column derivatisation with ninhydrin. After chromatographic separation, the ninhydrin–amino acid derivative eluted from the column was detected by absorbances at 570 and 440 nm (proline).

The determination of amino acids was made by comparing their retention times with those of a standard sample of seventeen amino acids (Supelco-Sigma-Aldrich, Alcobendas, Madrid, Spain): aspartic acid (Asp), glutamic acid (Glu), serine (Ser), alanine (Ala), arginine (Arg), cysteine (Cys), histidine (His), glycine (Gly), leucine (Leu), isoleucine (Ile), lysine (Lys), methionine (Met), threonine (Thr), phenylalanine (Phe), tyrosine (Tyr), valine (Val), and proline (Pro) (1 µm/mL). The determinations were performed in duplicate, and the results were expressed as nmol per sample.

Given the positive relationship observed in previous studies [15] between branched amino acids and certain parameters related to intestinal health, a separate calculation of BCAAs, AAAs, and their corresponding ratio was performed. Total branched-chain amino acids (BCAAs) were calculated as the sum of Leu, Ile, and Val; whereas total aromatic amino acids (AAAs) were the sum of Tyr and Phe.

### 2.3. Statistical Analysis

The dataset was analysed using a completely randomised design applying the General Linear Model (GLM) procedure in SAS (version 9; SAS Institute Inc., Cary, NC, USA). Comparative analysis of means was conducted using Duncan’s test (version 9; SAS Institute Inc., Cary, NC, USA). The diagnosed group was considered as a fixed effect according to the following model: Y_d_ = µ + α_d_, where Y_d_ represents the diagnosed group dependent variable, µ the overall mean, and α_d_ the effect of the diagnosed group. Given the wide range of ages and weights of the dogs, the possible relationship between these variables and the faecal amino acid profile was tested using Pearson’s correlation analysis (Statgraphics Centurion XIX, X64, version 19.2.01). Since no relationships were observed between animal weight or age and faecal amino acids, these variables were not included in the statistical model. Group means and their corresponding root mean square error (RMSE) were reported, with statistical significance set at *p* < 0.05. Pearson’s correlation analysis was performed to investigate potential relationships between faecal amino acid levels and CIBDAI (measured in Higueras et al. [16]) using Statgraphics (Statgraphics Centurion XIX, X64, version 19.2.01). For the linear discriminant analysis, the variables included in the model were using a stepwise algorithm (SAS STEPDISC), and the resulting model was executed and validated using the SAS DISCRIM procedure (version 9.4; SAS Institute Inc., Cary, NC, USA).

## 3. Results

**Figure 1:**
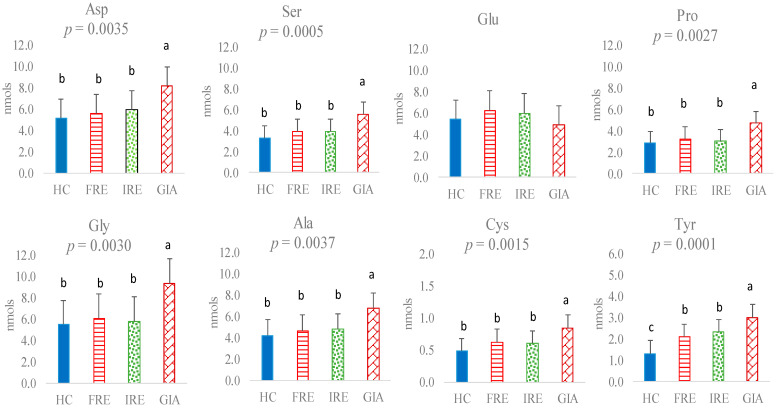
Non-essential amino acids (nmol) in faeces of healthy control (HC) dogs, food-responsive enteropathy (FRE) dogs, immunosuppressant-responsive enteropathy (IRE) dogs, and dogs parasitised with *Giardia* (GIA). Aspartic acid (Asp), serine (Ser), glutamic acid (Glu), proline (Pro), glycine (Gly), alanine (Ala), cysteine (Cys), tyrosine (Tyr). Values with different superscripts (a,b,c) are statistically significant.

**Figure 2:**
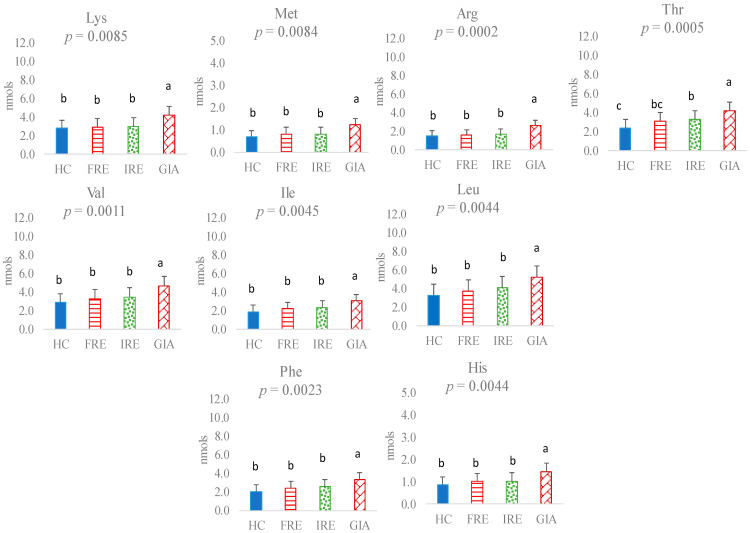
Essential amino acids (nmol) in faeces of healthy control (HC) dogs, food-responsive enteropathy (FRE) dogs, immunosuppressant-responsive enteropathy (IRE) dogs, and dogs parasitised with *Giardia* (GIA). Lysine (Lys), methionine (Met), arginine (Arg), threonine (Thr), valine (Val), isoleucine (Ile), leucine (Leu), phenylalanine (Phe), and histidine (His). Values with different superscripts (a,b,c) are statistically significant.

Concerning the faecal amino acid profile, no statistically significant differences were observed between the HC group and FRE or IRE dogs in levels of faecal Asp, Ser, Glu, Pro, Gly, Ala, Cys, Lys, Met, Arg, Val, Ile, Leu, Phe, or His (Figure 1 and Figure 2). However, HC presented lower faecal Tyr levels than FRE or IRE dogs (*p* = 0.0001) (Figure 1). The amino acid profile was not influenced by age or body weight, although body weight differed significantly between treatments (*p* = 0.0059). This was confirmed by the absence of a relationship between body weight and each of the amino acids (*p* > 0.05). Moreover, its inclusion as a covariate in the statistical model did not alter the results; therefore, it was ultimately excluded from the final analysis.

Additionally, the HC group had lower levels of Thr (*p* = 0.0005) compared to IRE dogs; however, no significant differences were found in faecal Thr amount between HC and FRE, whereas FRE dogs presented intermediate levels of Thr between HC and IRE (Figure 2).

In no case did the faecal amino acid levels between FRE and IRE dogs vary statistically. However, significant differences were observed between the GIA group and the other experimental groups for all amino acids except Glu. Specifically, the GIA group had higher faecal levels of Asp (*p* = 0.0035), Ser (*p* = 0.0005), Pro (*p* = 0.0027), Gly (*p* = 0.0030), Ala (*p* = 0.0037), Cys (*p* = 0.0015), Tyr (*p* = 0.0001), Lys (*p* = 0.0085), Met (*p* = 0.0084), Arg (*p* = 0.0002), Thr (*p* = 0.0005), Val (*p* = 0.0011), Ile (*p* = 0.0045), Leu (*p* = 0.0044), Phe (*p* = 0.0023), and His (*p* = 0.0044) (Figure 1 and Figure 2).

**Figure 3:**
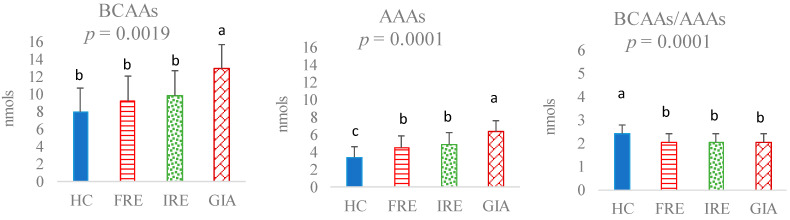
Branched-chain amino acids (BCAAs = sum of Leu, Ile, and Val), aromatic amino acids (AAAs = sum of Tyr and Phe), and the BCAAs/AAAs ratio in faeces of healthy control (HC) dogs, food-responsive enteropathy (FRE) dogs, immunosuppressant-responsive enteropathy (IRE) dogs, and dogs parasitised with *Giardia* (GIA). Values with different superscripts (a,b,c) are statistically significant.

Concerning BCAAs, no significant differences were observed between the HC, FRE, or IRE groups (Figure 3). However, the GIA group presented higher BCAA values than the other dog groups (*p* = 0.0019). On the other hand, AAAs showed more pronounced differences. Thus, the HC group was the one that presented the lowest AAA values compared to the other groups, while GIA had the highest (*p* = 0.0001). The FRE and IRE groups did not present significant differences between them in the AAA levels, generally presenting intermediate values between the HC and GIA groups. Consequently, the BCAAs/AAAs ratio was the highest in HC dogs compared to the rest of the other groups, which showed no significant differences among themselves (*p* = 0.0001).

Regarding CIBDAI, this was positively related to Tyr (r = 0.63; *p* = 0.0001), Thr (r = 0.48; *p* = 0.0001), Phe (r = 0.42; *p* = 0.0001), and AAAs (r = 0.55; *p* = 0.0001) and negatively related to the BCAAs/AAAs ratio (r = −0.27; *p* = 0.0134).

**Figure 4:**
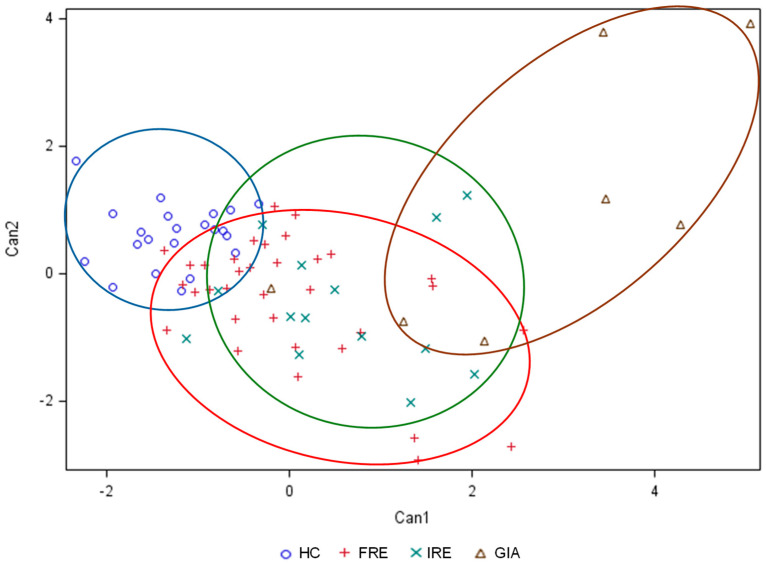
Linear discriminant analysis for faecal amino acids of healthy control (HC; dark blue) dogs, food-responsive enteropathy (FRE; red) dogs, immunosuppressant-responsive enteropathy (IRE; light green) dogs, and dogs parasitised with *Giardia* (GIA; brown).

The discriminant potential of the amino acid profile to differentiate between different enteropathies was evaluated through linear discriminant analysis (Figure 4). Multivariable analysis revealed that HC and GIA groups differed the most from the FRE and IRE groups, while the FRE and IRE groups overlapped to some extent.

The most influential variables contributing to canonical discriminant functions 1 and 2 were Tyr (*p* = 0.0001), Glu (*p* = 0.0003), Arg (*p* = 0.0001), and Phe (*p* = 0.1435).

Cross-validation results of the discriminant functions (Table 2) indicated that 44% of stool samples were not correctly assigned to their group, giving a 56% success rate. The HC group had the highest correct classification rate (100% correct assignments), followed by the GIA group (57%). However, 29% of GIA were misclassified as FRE and 14% as IRE. For the FRE group, 38% were assigned to the correct group, whereas 3% were misclassified as GIA, 27% as IRE, and 32% as HC. The IRE group had the lowest correct classification rate (29%), with 50% of stool samples classified as FRE, 14% as GIA, and 7% as HC.

## 4. Discussion

Few studies in veterinary medicine have evaluated the amino acid profile in the faeces of dogs with CIEs, and in some cases, the results have not followed similar trends. However, these comparisons have been limited to healthy dogs and a single group of sick animals, without including a broader evaluation of dogs with different types of enteropathies.

In the present study, no significant changes were observed in the essential or non-essential amino acid profiles of FRE and IRE dogs, nor in the total BCAAs or AAAs or the BCAAs/AAAs ratio. However, FRE and IRE dogs had higher levels of Tyr and total AAAs in faeces than healthy dogs. As previously mentioned, this is the first study to evaluate the amino acid profile of dogs with different enteropathies together, thereby providing novel information into the potential differences between these two groups of dogs based on their faecal amino acid profiles. Comparisons between sick animals with the HC group align with findings from previous studies in humans, which reported elevated Tyr levels in the faeces of individuals with IBD compared to healthy controls [20,21,22]. Tyr is an AAA obtained from Phe via phenylalanine hydroxylase in the liver, which is further metabolised into neurotransmitters, such as dopamine, norepinephrine, and adrenaline, as well as melanin. Impaired conversion of Phe to Tyr could result in reduced cognitive performance [23], and alterations in the Phe/Tyr ratio have also been observed in inflammatory conditions [24]. Furthermore, it is interesting to note that Tyr can give rise to the compound *p*-cresol [25] by the action of certain anaerobic microorganisms, a compound that is genotoxic to colonocytes and reduces mitochondrial oxygen consumption in these cells [26]. In addition, other metabolites such as *p*-cresyl sulfate can be synthesised from *p*-cresol in the host mucosa [26], subsequently entering circulation or being excreted in urine [27]. These metabolites have been associated with increased oxidative stress and, in some cases, cell death [27]. Excessive levels of such compounds have also been linked to reduced gut microbiota diversity [28]. Other investigations have reported significant changes in other faecal AAAs such as Phe or Trp. Thus, Marchesi et al. [20] observed a greater abundance of Phe in faecal samples from patients with various CIEs. Conversely, Higueras et al. [15] reported a reduced proportion of Phe in faecal samples from sick animals compared to the control group, whereas this amino acid was found to be increased in blood. More numerous are the authors who have identified alterations, primarily increases in the aromatic amino acid Trp, in both humans and animals [14,21,22], with evidence linking microbial Trp metabolites to gut disorders [29]. The discrepancies between studies regarding the presence of amino acids in faeces may be due to factors related to the sample heterogeneity across investigations, as well as methodological differences, including whether prior hydrolysis was employed in the analytical process [15].

It is interesting to note that although the levels of Tyr, Phe, and AAAs were not different between FRE and IRE dogs, a direct relationship was observed with the severity index. In other studies, IRE dogs have also shown more severe signs of disease [18], although based on the levels of these AAs in faeces and with the methodology used for their measurement, it would not be possible to predict what treatment an individual CIE dog would respond to based on faecal AAs analysis. On the other hand, neither the total BCAA levels nor the BCAAs/AAAs ratio were significantly affected between FRE, IRE, and control groups in the present study. Higueras et al. [15] did not observe any significant changes in these AAs in a previous study involving FRE dogs compared to a control. However, some of the BCAAs, such as Leu, showed a direct relationship with indicators of intestinal health, such as the presence of short-chain fatty acids (SCFAs) in faeces. This relationship was also observed in the present study (data not presented), confirming the previous results. It has been reported that BCAA catabolism contributes to the synthesis of all fatty acids [28]. Specifically, Val and Ile contribute to the lipogenic propionyl-CoA pool, which acts as a primer for fatty acid synthase in the production of odd-chain fatty acids [30]. Moreover, the catabolism of AAAs by the gut microbiome has revealed that the derived metabolites are bioactive compounds that may exert varying effects on the gut and other organs [29]. Thus, under certain environmental conditions, such as changes in the gut pH or other factors associated with gut diseases, a dysregulation of the microbiome may occur, altering metabolite production [29].

Another remarkable result of the present study was the higher Thr content in the faeces of IRE dogs compared to control dogs, with FRE dogs showing intermediate values. In children with ulcerative colitis, Kolho et al. [31] observed elevated levels of Thr in faecal metabolic fingerprinting compared to controls. Moreover, an in-depth metabolomics study by Filmoniuk et al. [32] on children with IBD found significant changes in the metabolism of 16 compounds. Among these, the metabolism of Thr was notably affected, followed by changes in the metabolism of Phe and its derived products, which aligns with the results of the present study. In fact, in the present study, Thr had a significant direct relationship with the severity index, as did Tyr and Phe. Thr is an essential amino acid that serves as a substrate for mucin synthesis [33] and plays a role in lipid metabolism regulation [34]. The importance of Thr in mucus formation is such that, even when Thr supply is deficient, other tissue growth functions may be limited while mucin production is maintained [35]. Thus, Thr is critical for barrier function and gut homeostasis and may influence the intestinal immune system [36]. The higher levels of this amino acid found in the faeces of sick animals in the present study could be explained by its preferential use by mucosal cells for mucin synthesis when present in the intestinal lumen [37]. In addition, it has been reported that under intestinal inflammation, Thr uptake is enhanced by intestinal cells for mucus production, contributing to gut protection [38].

In relation to the group of dogs infected with *Giardia* that also experienced gastrointestinal problems, they presented the highest levels of faecal amino acids, including Thr and Tyr, compared to the other groups. Additionally, these animals presented the highest faecal levels of most amino acids (except Glu), as well as AAAs, and BCAAs in comparison to the HC, FRE, and IRE groups. *Giardia* spp. is a protozoan parasite that infects humans and other vertebrates, residing in the upper small intestine and causing severe diarrhoea, malabsorption, and other gastrointestinal diseases worldwide [39]. This parasite has been described to utilise amino acids differently [40] and to produce secretory/excretory proteins linked to infection [41]. It also induces structural damage that compromises epithelial integrity and function [42]. Additionally, some studies indicate that *Giardia* proteins secreted upon contact with epithelial cells may facilitate colonisation of the host’s small intestine and deactivate host innate immune factors, such as nitric oxide production [43]. These changes might explain the increased presence of AAs in the faeces of GIA dogs. It is important to note that many GIA infections are asymptomatic and have even been considered to protect against other gastrointestinal processes or resolve spontaneously [42]. However, this infection can also lead to irritable bowel syndrome and other intestinal disorders [42], making its differentiation from other digestive conditions essential. According to the results of the present study, the faecal amino acid profile could serve as a non-invasive tool complementary to existing diagnostic techniques for this infection.

It is interesting to note that in general the AAs that presented the greatest discriminating power between the different groups were Tyr, Phe, Glu, and Arg. The differences in Tyr and Phe between the groups of dogs have been previously discussed, with Tyr, derived from the metabolism of Phe, showing the most significant variation. However, Glu and Arg levels did not significantly differ among the groups, except in GIA dogs, which presented higher Arg levels compared to the others. Arg is a semi-essential amino acid that serves as a precursor for the synthesis of proteins, nitric oxide (NO), creatinine, and urea [44]. Einarsson et al. [42] reported that Giardia trophozoites use the arginine dihydrolase pathway for energy production and that the arginine deiminase is one of the metabolic enzymes secreted by the parasite upon interaction with the epithelium. Giardia-induced Arg depletion is a mechanism to prevent the production of NO by the host since this compound is toxic to the parasite [42,43]. Interestingly, Arg can be synthesised from glutamine [45]. The fact that Glu was the only faecal AA that did not show higher levels in the GIA group compared to the other experimental groups could indicate its increased consumption for Arg synthesis. Glutamate is a non-essential amino acid that can be synthesised in the body and serves as an important energy source for the proliferation of intestinal lymphocytes and epithelial cells [46]. Consequently, it plays significant roles in the maintenance of mucosal structure, tight junctions, and mucosal permeability [47]. In a metabolomic study of individuals with IBD, Filimoniuc et al. [32] also highlighted the metabolism of Glu and Arg being most influential in relation to the presence of disease, along with the metabolism of Thr or Phe [32]. Additionally, patients with IBD often exhibit Arg deficiency and alterations in the Arg metabolic pathway [48]. Both Glu and Arg, have beneficial effects on the modulation of inflammatory cytokines, which may influence gut integrity [49].

Despite the discriminating potential of some of the amino acids mentioned, the faecal AA analysis could not accurately distinguish between the FRE and IRE dogs, as both groups showed considerable overlap. Therefore, based on the results, we could not say that the two groups have distinct amino acid metabolism. Furthermore, although the GIA dogs had a markedly different AA profile compared to the other groups, the statistical model built to distinguish between groups did not accurately differentiate this group from FRE or IRE.

## 5. Conclusions

In conclusion, dogs infected with GIA showed significantly greater faecal amino acid excretion compared to the other groups. Additionally, healthy dogs presented some significant differences in their faecal amino acid profiles compared to dogs with FRE or IRE, particularly in Thr and AAAs (especially Tyr). However, the faecal amino acid profile did not differ between dogs with FRE and IRE. Therefore, it would not be possible to predict an individual CIE-affected dog’s response to treatment based on faecal AA analysis. Further studies are necessary to better understand the different responses to dietary treatment in these two groups of dogs.

## Figures and Tables

**Table 1:** Composition of diets (averaged g/100 g according to manufacturer’s composition) prior to the dietary change of healthy control dogs (HC), food-responsive enteropathy dogs (FRE), immunosuppressant-responsive enteropathy dogs (IRE), and dogs parasitised with *Giardia* (GIA).

Variable	HC (*n* = 22)	FRE (*n* = 35)	IRE (*n* = 18)	GIA (*n* = 9)
Humidity	9.50 ± 0.71	8.38 ± 0.48	8.50 ± 0.49	8.50 ± 0.50
Crude protein	23.05 ± 4.68	23.71 ± 4.50	23.73 ± 5.07	22.70 ± 4.90
Crude fat	13.78 ± 4.10	14.87 ± 4.30	13.46 ± 4.21	13.54 ± 4.41
Crude fibre	2.83 ± 0.78	2.83 ± 1.89	2.12 ± 1.63	2.57 ± 1.01
Crude ash	7.64 ± 1.20	6.70 ± 0.87	5.83 ± 1.00	6.23 ± 2.07
Nitrogen-free extractives	43.18 ± 7.91	43.49 ± 7.42	46.34 ± 8.36	46.44 ± 8.20
Calcium	1.47 ± 0.16	1.09 ± 0.30	0.91 ± 0.13	1.43 ± 0.06
Phosphorus	1.03 ± 0.17	0.77 ± 0.21	0.64 ± 0.09	0.92 ± 0.10
Sodium	0.30 ± 0.08	0.37 ± 0.08	0.35 ± 0.07	0.40 ± 0.08
∑n-3	0.67 ± 0.47	1.03 ± 0.89	2.00 ± 1.83	0.55 ± 0.35
Metabolic energy (kcal/kg) ^1^	3583 ± 193	3711 ± 201	3742 ± 163	3684 ± 221

^1^ Calculated according to NRC [19].

**Table 2:** Classification accuracy (%) based on faecal amino acid profile of healthy control (HC) dogs, food-responsive enteropathy (FRE) dogs, immunosuppressant-responsive enteropathy (IRE) dogs, and dogs parasitised with *Giardia* (GIA) (% assigned correctly) according to discriminant functions (cross-validation).

	FRE	GIA	IRE	HC	TOTAL (%)
FRE	38.24	2.94	26.47	32.35	100
GIA	28.7	57.14	14.29	0.00	100
IRE	50.00	14.29	28.57	7.14	100
HC	0.00	0.00	0.00	100.00	100

## Data Availability

Data are contained within the article.

## Abbreviations

The following abbreviations are used in this manuscript:

AA	amino acid
AAA	aromatic amino acid
Ala	alanine
Arg	arginine
Asp	aspartic acid
BCAA	branched-chain amino acid
BCS	body condition score
CCECAI	canine chronic enteropathy clinical activity index
CIE	chronic inflammatory enteropathy
CIBDAI	canine inflammatory bowel disease activity index
CSIC	Spanish National Research Council
CVMTH	Complutense Veterinary Medicine Teaching Hospital
Cys	cysteine
DNA	deoxyribonucleic acid
FRE	food-responsive enteropathy
GIA	*Giardia* infection
Glu	glutamic acid
Gly	glycine
HC	healthy control
His	histidine
HCl	hydrochloric acid
Ile	isoleucine
IBD	inflammatory bowel disease
IRE	immunosuppressant-responsive enteropathy
Leu	leucine
Lys	lysine
Met	methionine
MCS	muscle condition score
NaOH	sodium hydroxide
Phe	phenylalanine
Pro	proline
RMSE	root mean square error
Ser	serine
Thr	threonine
TLI	trypsin-like immunoreactivity
Trp	tryptophan
Tyr	tyrosine
Val	valine
